# Catalytic Activity of Thermolyzed [Co(NH_3_)_6_][Fe(CN)_6_] in CO Hydrogenation Reaction

**DOI:** 10.3390/molecules26133782

**Published:** 2021-06-22

**Authors:** Alevtina N. Gosteva, Mayya V. Kulikova, Yulya P. Semushina, Mariya V. Chudakova, Nikita S. Tsvetov, Vasilii V. Semushin

**Affiliations:** 1 I.V. Tananaev Institute of Chemistry and Technology of Rare Elements and Mineral Raw Materials—Subdivision of the Federal Research Centre «Kola Science Centre of the Russian Academy of Sciences» Science Centre of Russian Academy of Sciences, «Academic town», 26a., Apatity, 184209 Murmansk, Russia; semushinajp@mail.ru (Y.P.S.); n.tsvetov@ksc.ru (N.S.T.); semushinvv@mail.ru (V.V.S.); 2 A.V. Topchiev Institute of Petrochemical Synthesis, RAS, Leninsky prospect, 29, 119991 Moscow, Russia; m_kulikova@ips.ac.ru (M.V.K.); Chudakova@ips.ac.ru (M.V.C.)

**Keywords:** double complex salt, thermal decomposition, CO hydrogenation

## Abstract

Currently, the processes of obtaining synthetic liquid hydrocarbons and oxygenates are very relevant. Fischer-Tropsch synthesis (FTS) is the most important step in these processes. The products of thermal destruction in argon of the mixture [Co(NH_3_)_6_][Fe(CN)_6_] and Al(OH)_3_ were used as catalysts for CO hydrogenation. The resulting compositions were studied using powder X-ray diffraction, IR spectroscopy, elemental analysis, SEM micrographs. The specific surface area, pore and particle size distributions were determined. It was determined that the DCS-based catalysts were active in the high-temperature Fischer-Tropsch synthesis. The effect of aluminum in the catalyst composition on the distribution of reaction products was revealed.

## 1. Introduction

Fischer-Tropsch synthesis (FTS) is catalyzed by Group VIII metals (iron, cobalt, nickel). In the presence of cobalt-based catalysts, yields of products and formation of long-chain alkanes are strongly dependent on the nature of the catalyst support [1]. The yield of olefins’ and oxygenates’ high activity in the water shift reaction are significant on iron catalysts [2]. To obtain systems that have the advantages of both metals, catalysts with bimetallic iron-cobalt containing particles are created. In [3] the effect of the nature of the substrate (oxide and carbon carriers) on the composition of the products obtained is shown. In [2] the formation of an alloy of metals was found, which enhances the ability to incorporate CO, which leads to an increase in the selectivity for alcohols. It was found [4], that the supported Fe-Co bimetallic catalysts showed higher activity in comparison with catalysts from pure metals. In [5] it was found, that joint impregnation leads to the formation of mixed Fe-Co oxides, the reduction of which leads to the formation of alloys. It was also found that the introduction of an increase in the cobalt content in the system accelerates the hydrogenation of carbon monoxide, and the formation of mixed particles of cobalt and iron promotes the formation of light olefins in comparison with the monometallic cobalt catalyst, and a decrease in activity during the Fischer-Tropsch synthesis. In [6], it is shown that the addition of PdTe and PdTe_1−x_Bi_x_ to the standard cobalt catalyst increases both yield and selectivity with respect to the liquid phase and is the shift in the conversion products toward the diesel and wax fractions.

When operating catalysts for the CO conversion, a necessary stage is the preliminary activation of the catalyst in a stream of hydrogen, CO, or synthesis gas at elevated temperatures. Usually, this process takes place in a special apparatus and then the reduced catalyst is transferred to the reactor. This is complicated by the fact that the catalysts are pyrophoric and this procedure should be carried out very carefully. The creation of catalytic systems that do not require an activation stage is highly relevant.

Previously, the authors of the article studied the methods of forming active catalytic systems for CO hydrogenation without a preliminary activation step. For example, in [7,8] the catalysts were formed by the method of organic matrixes with further IR-pyrolysis of a joint metal salt (iron/cobalt) and various nature polymer solution. It was found that the nature of the polymer significantly affects the selectivity and composition of the resulting products. In the presence of Fe-copolymer of styrene and divinylbenzene, the C_5+_ hydrocarbon selectivity was reached 60–80%, while an increased content of normal paraffins is observed. And in the case of Co-polyvinylalcohol, the selectivity for methane exceeds 70% with a CO conversion of 100%.

The solid decomposition products of double complex salts (DCS) exhibit promising catalytic properties. The research group of Potemkin D.I. is engaged in the systematic research of catalysts for total and preferential CO oxidation. We will consider this reaction further. These authors frequently use DCS as precursors for catalysts, which include one 3d-metal and one platinum metal. Often, catalysts obtained by thermolysis of DCS in various gas atmospheres are more efficient than catalysts that consist only of platinum metals. In [9], nanopowders of unordered solid solution Pt_0.5_Co_0.5_ (thermolysis product of [Pt(NH_3_)_4_][Co(C_2_O_4_)_2_(H_2_O)_2_]∙2H_2_O), were more active in CO preferential oxidation than Pt (calcination product of [Pt(NH_3_)_4_](NO_3_)∙2H_2_O). In the [10] article, the thermal destruction product [Au(en)_2_]_2_[Cu(C_2_O_4_)_2_]_3_∙8H_2_O was investigated as a CO oxidation catalyst in the realistic hydrogen rich mixture containing CO_2_ and H_2_O. Au_0.4_Cu_0.6_/CeO_2_ showed better performance with higher CO consumption at higher selectivity compared to Au/CeO_2_ catalyst. The authors also investigated the effect of the support—γ-Al_2_O_3_, SiO_2_, CeO_2_ on the catalytic activity. In the work shown on [11] the effect of metal replacement in the anion on the catalytic activity during thermolysis of precursors from DCS [Pt(NH_3_)_4_][Ni(C_2_O_4_)_2_(H_2_O)_2_]∙2H_2_O, [Pt(NH_3_)_5_Cl][Fe(C_2_O4)_3_]∙4H_2_O, [Pt(NH_3_)_4_][Co(C_2_O_4_)_2_(H_2_O)_2_]∙2H_2_O investigated. The activity of the catalysts decreases in the order Pt_0.5_Co_0.5_ > Pt_0.5_Ni_0.5_ > Pt_0.5_Fe_0.5_ >> Pt.

In the work shown on [12], it was found that the active phase of the reduction product of the [Co(NH_3_)_6_][Fe(CN)_6_]+Al(OH)_3_ mixture consists of the CoFe intermetallic compound. It changes the course of the Fischer-Tropsch synthesis reaction and alkenes become the main product (>90%). The aim of this work is to study the thermolysis products of [Co(NH_3_)_6_][Fe(CN)_6_] in argon as catalytic compositions for the FTS process. The effect of the addition of Al(OH)_3_ to [Co(NH_3_)_6_][Fe(CN)_6_] is shown by the example of changes on thermal behavior, X-ray phase composition of thermolysis products, and, consequently, catalytic activity.

## 2. Results and Discussion

### 2.1. Characterization of Catalytic Compositions

DCS [Co(NH_3_)_6_][Fe(CN)_6_] and a mixture of aluminum hydroxide and DCS were precursors for the preparation of catalytic compositions. STA curves for Al(OH)_3_, [Co(NH_3_)_6_][Fe(CN)_6_] and their mixtures were obtained for a more complete picture of the thermal decomposition of the mixed catalytic composition and elucidation of the possible mutual influence of the precursor components.

**Figure 1:**
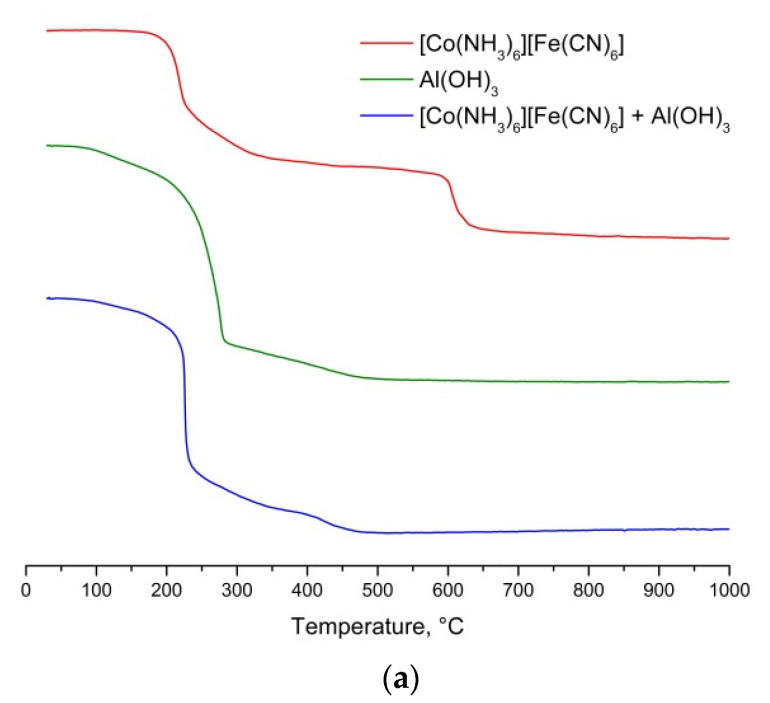

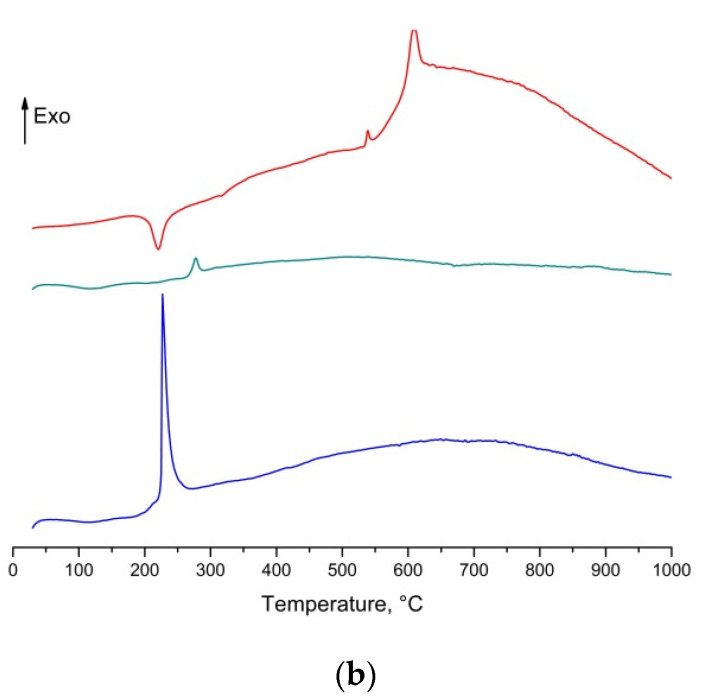
Thermal analysis curves for [Co(NH_3_)_6_][Fe(CN)_6_], Al(OH)_3_ and mixtures thereof: (**a**) TG, (**b**) DSC.

Thermal decomposition of Al(OH)_3_ was characterized by three stages of weight loss (Figure 1): 60–140 °C (4 wt.%—removal of sorption water occurs); 140–290 °C (48 wt.%) and 290–550 °C (10 wt.%). The last two weight losses were associated with the dehydration of Al(OH)_3_. X-ray amorphous products were obtained by calcining Al(OH)_3_ at 350 and 550 °C. A slightly crystallized γ-Al_2_O_3_ was obtained by calcining at 900 °C. The endothermic peak of the evaporation of sorbed water, a relatively large exothermic peak of decomposition and, presumably, a weakly pronounced endothermic peak before the main stage of decomposition can be seen on the DSC curve. Weight loss in the range 290–550 °C was not accompanied by a pronounced peak.

Thermal decomposition of [Co(NH_3_)_6_][Fe(CN)_6_] was characterized by the following five main stages of weight loss in the temperature ranges 120–230 (22 wt.%), 230–350 (12 wt.%), 350–450 (2 wt.%), 450–550 (1 wt.%) and 550–800 °C (16 wt.%) (Figure 1). The decomposition processes of DCS were usually multistage and are characterized by several parallel processes of decomposition of ligands [13]. According to DSC data, the first, fourth, and fifth stages of decomposition were characterized by endothermic and two exothermic peaks. The processes, that were occurring in the temperature range 230–550 °C, did not have significant heat effects.

The thermal behavior of a mixture of Al(OH)_3_ and [Co(NH_3_)_6_][Fe(CN)_6_] was characterized by the following four main stages, in temperature ranges similar to the individual components: 60–140 °C (2.5 wt.%, this is similar Al(OH)_3_), 140–230 °C (41 wt.%), 230–350 °C (12 wt.%) and 350–550 °C (6 wt.%) (Figure 1). Weight loss stopped after 550 °C. However, the heat effects of the processes occurring during the thermolysis of the mixture were very different from the individual components. One distinct exothermic peak could be found on the DSC curve, corresponding to the main process of weight loss in the range 140–230 °C. Note that, based on the DSC data, the position of the peaks corresponding to the steps of mass loss changes and the sign of the heat effect changes. The decomposition of the mixture ended earlier than the DSC itself. A significant change in the mechanism of decomposition of DCS in the presence of Al(OH)_3_ occurs.

The choice of temperatures for obtaining catalytic compositions was due to the following factors. First, according to the literature it was determined [14,15] that the formation of the CoFe intermetallic compound during thermolysis in argon [Co(NH_3_)_6_][Fe(CN)_6_] occurs at temperatures of 600–675 °C. Therefore, in this work, the average temperature was chosen in the indicated range, 650 °C.

Secondly, as it was written above, in the work of [12], promising data were obtained on catalysis in the FTS reaction. The composition [Co(NH_3_)_6_][Fe(CN)_6_] + Al(OH)_3_ was used as a precursor for the catalyst, the calcination temperature in hydrogen was 380 °C. Since the same composition was also used in this study, a similar temperature was chosen, namely, 350 °C.

The characteristics of the investigated catalytic compositions were shown in Table 1. Average values were given, each parameter was determined at least 3 times.

Diffraction patterns of samples **I** and **II** are shown in Figure S1: Powder X-ray diffraction patterns of [Co(NH_3_)_6_][Fe(CN)_6_] and catalytic compositions. No characteristic bands for NH_3_ and CN^−^ were found in the IR spectra of samples **I** and **II** (Figure S2: IR spectra of [Co(NH_3_)_6_][Fe(CN)_6_] and catalytic compositions). Consequently, ligands were not present in unchanged/slightly altered forms in the composition of thermolysis products.

**Figure 2:**
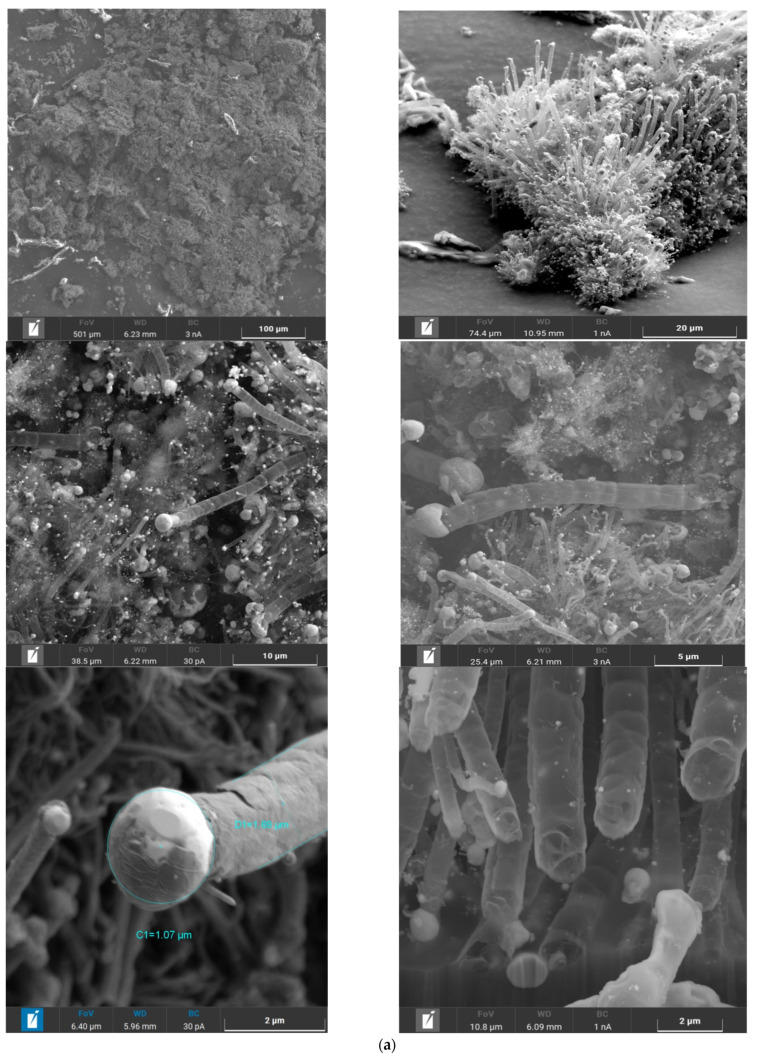

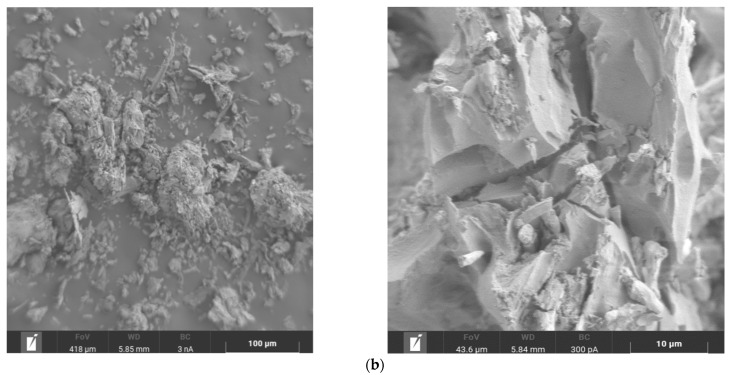
SEM micrographs of thermolysis productsof samble **I**—(**a**), for samble **II**—(**b**).

Photomicrographs of catalytic compositions were shown in Figure 2.

According to microprobe analysis, it was determined that sample **I** is a hollow carbon tube. A ball of intermetallic compound Co_0.5_Fe_0.5_ was located at the end of these tubes. Sample **II** had a powdery structure without a definite appearance. Sample **II** contains a homogeneous mixture of Co, Fe, and Al.

The distribution of particles and pores by size was given in Table 2 and Table 3. Mixing DCS with Al(OH)_3_ led to an increase in the size of the main part of particles in thermolysis products from 4 to 27 μm. This can be explained by the presence of a larger amount of aluminum particles in the sample. Based on Figure 2, it can be seen, that in the case of sample **I**, the main part of the specific surface area was formed by hollow carbon tubes, and in sample **II**, by aluminum particles.

### 2.2. Catalytic Tests

It could be seen (Table 4) that the thermal destruction products DCS and DCS + Al(OH)_3_- catalysts are active in the CO hydrogenation reaction without a preliminary activation step. With an increase of the synthesis temperature, a raise in the conversion of CO is observed on both catalysts: up to 93% for **I** and up to 35% for **II**. A high formation of by-products, such as gaseous hydrocarbons, with a selectivity of up to 61%, is observed on the catalyst without aluminum. The specific activity (A, molCO·gMe^−1^ c^−1^) of the catalysts also rises with increasing temperature, while the values are higher for the **II** catalyst, especially in the region of 290–310 °C. The selectivity for oxygenates was low and did not exceed 10%. On a catalyst containing aluminum, a higher selectivity for the target C_5+_ hydrocarbons was observed.

In the works of [16,17,18,19], they used different carbonaceous materials such as activated carbon, carbon nanofiber, carbon nanotube and carbon spheres. On these systems, the selectivity for C_5+_ was 40–80% with a CO conversion of 5–60%. It should be noted that all catalytic systems were preliminarily reduced in a stream of hydrogen for 20 h at temperatures of 350–400 °C. Whereas the catalysts formed in this work exhibited high activity in CO hydrogenation without a preliminary activation step. This makes them extremely promising for further study.

**Figure 3:**
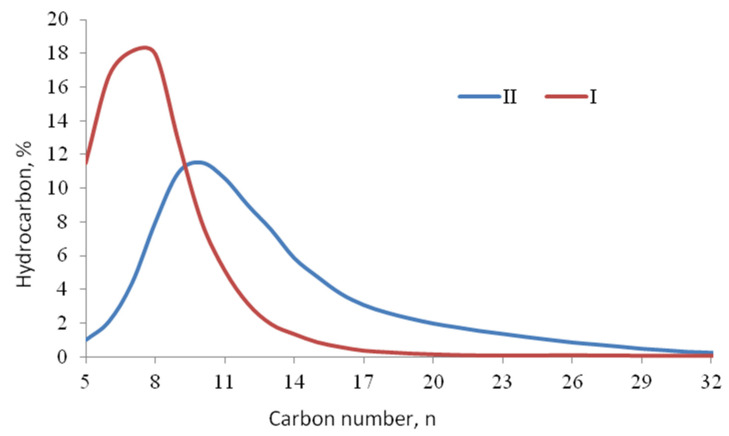
Hydrocarbon distribution on **I** and **II** catalysts at optimal FTS temperature.

Figure 3 shows that the distribution of the products of the Fischer-Tropsch synthesis on catalysts was significantly different. Thus, on the **I** catalyst, the maximum hydrocarbon distribution was a hydrocarbon with a chain length of 6–8 carbon atoms and the addition of aluminum to the catalyst composition shifts the maximum to C_9_–C_10_. It was also worth noting that on the **II** sample, more intense formation of higher molecular weight synthesis products occurs: the content of C_19+_ hydrocarbons reaches 15%, while on the **I** catalyst it does not exceed 5%. This is because the introduction of aluminum into the system leads to the formation of weak Lewis centers, which are responsible for the polymerization activity of the Fischer-Tropsch synthesis catalyst, which leads to the formation of hydrocarbons with a longer chain length in all fractions [20].

On the other hand, the composition of the products is influenced by the pore size distribution. From the data in Table 2 and Table 3, the porosity of sample **I** is higher than for sample **II**. Most likely, the smaller pores of sample II increase internal diffusion hindrances during the reaction, which leads to the formation of hydrocarbons with a longer chain length. The larger pores of the sample favor a lighter hydrocarbon product formation due to a decrease in internal diffusion hindrances [21].

From the data in Table 4, it could be seen that the introduction of aluminum into the composition of the catalyst leads to a twofold decrease in the content of olefins in the products. Most likely, this was due to an increase in the fineness of cobalt due to the presence of aluminum in the catalyst composition and the contribution of cobalt to the bimetallic catalyst became more significant, which led to an increase in the yield of hydrocarbons of normal structure and a decrease in the yield of olefins [22].

Authors of [23] found that an increase in the porosity of the support leads to a higher degree of metal reduction during the Fischer-Tropsch synthesis, which contributes to a more significant formation of olefins, which is in good agreement with the data in Table 5.

**Figure 4:**
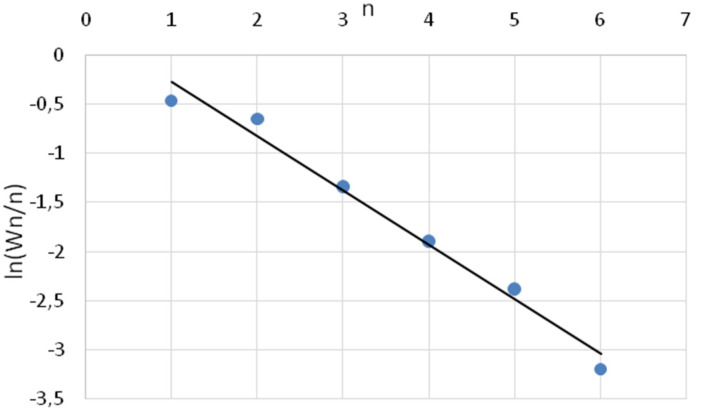
Typical Anderson-Schulz-Flory pattern for alcohol distribution.

Alcohols, as can be seen from Figure 4, fit quite well on a straight line, which indicated that, most likely, oxymethylene radicals were formed on the surface of the catalyst, followed by polycondensation with the formation of alcohols [24]. This type of mechanism was typical for chain growth on cobalt Fischer-Tropsch synthesis catalysts [20].

## 3. Materials and Methods

### 3.1. Materials

DCS [Co(NH_3_)_6_][Fe(CN)_6_] was obtained by stoichiometric mixing of equivalent amounts of solutions of the starting cationic and anionic precursor complex. [Co(NH_3_)_6_]Cl_3_ (Vekton, Russia), K_3_[Fe(CN)_6_] (Neva Reaktiv, Russia) with less than 10^−5^ mol% impurities were used. Powder X-ray diffraction (XRD), IR spectroscopy, elemental analysis results are the same as described in [14,15,25,26,27]. The curves of thermal analysis in argon, which were obtained in this study and reported in [14,26], have a similar character.

### 3.2. Obtaining Catalytic Compositions

The sample **I** was the product of thermal destruction of [Co(NH_3_)_6_][Fe(CN)_6_] in argon at 650 °C. Sample **II** was obtained by mixing [Co(NH_3_)_6_][Fe(CN)_6_] with freshly precipitated Al(OH)_3_. The resulting mixture was dried to constant weight and calcined in argon at 350 °C.

### 3.3. STA Measurements

Simultaneous thermal analysis was made using the equipment STA 409 PC Luxx (NETZSCH-Gerätebau GmbH, Selb, Germany, Selb, 2009). Experiments were provided in a dynamic atmosphere of argon. The heating rate was 10 °C·min^−1^, the temperature range was 30–1000 °C. Later, 10 mg of the sample was placed in Al_2_O_3_-crucible.

### 3.4. Isothermal Experiments

Thermal decomposition of the catalytical mixtures under isothermal conditions was carried out in a tubular reactor in a stream of Ar. A sample of ~0.5 g was heated to a prescribed temperature in a quartz boat with a heating rate of 10 °C/min and was held at this temperature for 1 h. After the end of the soaking time, the reaction tube was removed from the furnace and cooled to room temperature in a stream of Ar. The tubular muffle furnace Nabertherm RT 50-250/11 (Nabertherm GmbH, Lilienthal, Germany, 2013) was used. Argon was pre-dried by passing through concentrated H_2_SO_4_, the flow rate was 12–15 L·h^−1^.

### 3.5. Physicochemical Research Methods

IR spectra were recorded on a Nicolet 6700 FT-IR spectrophotometer (Thermo Fisher Scientific Inc., Hillsboro, OR, USA, 2010) in the 4000–400 cm^−1^ region (KBr tablets). X-ray phase analysis of products of thermal destruction of DCS was made in 2*θ* range 5°–90° on a Shimadzu XRD 6000 powder diffractometer (Kyoto, Japan, 2008) equipped with a Cu-Ka source (*λ* = 1.5418 Å) and graphite monochromator for the diffracted beam. Indexing of the diffraction patterns was done using data for pure metals and compounds reported in the JCPDS-ICDD PDF4+ database (2019). The porous structure of the samples was studied by the method of low-temperature sorption of nitrogen on a Tristar 3020 instrument (Norcross, GA, USA, 2009). Pore size distribution was determined on the device Shimadzu Sald-201V (Kyoto, Japan, 2007), refractive index *n* = 1.65 − 0.2i. Elemental analysis was carried out using the analyzer ELTRA-2000 (Alpha Resources, LLC, Stevensville, MI, USA, 2004) and Thermo Flash 2000 (Loughborough, Great Britain, 2010).

### 3.6. Sample Preparation for Micrographs

The samples were placed on an aluminum table. The table was covered with non-sprayed conductive carbon tape. TESCAN AMBER (Brno, the Czech Republic, 2021)—a two-beam scanning electron microscope based on a field emission cathode of the Schottky type with an additional Orage ion column with a Ga ion source, was used. Energy dispersive detector EDS Ultime 100 manufactured by Oxford Instruments (High Wycombe, England, 2021) was applied.

### 3.7. Catalytic Tests

The synthesis of hydrocarbons from CO and H_2_ was carried out in a flow catalytic unit with a fixed-bed reactor. The catalyst was diluted with an equal volume of quartz to reduce the resistance of the powder layer to gas flow and placed on the quartz layer to prevent the material from spilling out of the reaction zone.

The synthesis was carried out under continuous operation using synthesis gas CO:H_2_ = CO:H_2_ = 1:1 (mol.) at a pressure of 2 MPa and gas hourly space velocity of 300 h^−1^. The temperature rise in the range of 210–330 °C was carried out stepwise (by 20 °C every 12 h). 12 h of the experiment are required to enter the mode and to accumulate the necessary amount of reaction products for analysis. At the end of each isothermal regime, gas and liquid samples were taken for analysis.

### 3.8. Analysis of Reagents and Reaction Products in FTS

The initial synthesis gas and gaseous products of synthesis were analyzed by gas chromatography on a Krystallux-4000 chromatograph (Russia, Yoshkar-Ola, 2017) with a TCD detector and the carrier gas is helium. Two chromatographic columns were used. A column filled with CaA molecular sieves (3 m × 3 mm) was used to separate CO and N_2_. Temperature condition: isothermal, 80 °C. To separate CO_2_ and C_1_–C_4_ hydrocarbons a column filled with Haye Sep R (3 m × 3 mm) was used. Temperature mode: programmed, 80–200 °C, 8 °C min^−1^.

The mixture of liquid hydrocarbons was analyzed by gas-liquid chromatography on a Kristallux-4000M chromatograph (Russia, Yoshkar-Ola, 2017). The detector is flame ionization. Gas flow rate: nitrogen −30 mL min^−1^, hydrogen −25 mL min^−1^, air −250 mL min^−1^. OV—351 capillary column (50 m × 0.32 mm). Temperature mode: programmed, 50 °C (2 min) −50–260 °C, 6 °C min^−1^ −260–270 °C, 5 °C min^−1^ −270 °C (10 min).

Oxygen-containing products in the aqueous phase were analyzed by gas-liquid chromatography on a Kristallux-4000M chromatograph (Russia, Yoshkar-Ola, 2017). The detector was flame ionization. Gas flow rate: helium −20 mL min^−1^, hydrogen −25 mL min^−1^, air −250 mL min^−1^. An HP-FFAP capillary column (Nitroterephthalic Modified Polyethylene Glycol) (50 m × 0.32 mm × 0.50 μm) was used for analysis. The sample volume is 0.3 μL. Temperature mode: programmable, 70 °C (8 min), 70 °C–110 °C, 10 °C min^−1^, 110 °C–220 °C, 15 °C min^−1^ −220 °C (10 min). For quantitative calculation, the internal standard method was used (the standard is isobutyl alcohol).

The following indicators were used to evaluate the activity of the catalyst: CO conversion (percentage of the mass of reacted carbon monoxide to the mass of CO entering the reaction zone), product yield (number of grams of the product obtained by passing 1 m^3^ of synthesis gas through the catalyst reduced to normal conditions), activity (A)—the number of moles of CO that reacted per 1 g of the sum of metals in 1 s, selectivity (S)—the percentage of carbon used to form product related to the total amount of carbon reacted.

## 4. Conclusions

Intermetallic compound Co_0.5_Fe_0.5_ and X-ray amorphous carbon in the form of hollow tubes is obtained by thermal decomposition in argon [Co(NH_3_)_6_][Fe(CN)_6_]. Oxides CoFe_2_O_4_, CoO, Fe_3_O_4_ are formed during the thermal destruction of a mixture of [Co(NH_3_)_6_][Fe(CN)_6_] and Al(OH)_3_. It was shown that the synthesized DCS-based catalysts are active in the high-temperature Fischer-Tropsch synthesis. It was found that the introduction of aluminum into the composition of the catalyst leads to the formation of higher molecular weight hydrocarbons and a decrease in the content of olefins in the reaction products. This is most likely associated with the formation of a more dispersed cobalt phase on the one hand and the formation of weak Lewis centers, which are responsible for the polymerization activity of the catalyst on the other hand.

## Figures and Tables

**Table 1:** Characterization of catalytic compositions.

Precursor	T, °C	No. Sample	Residue from Calcination, wt.%	Co, wt.%	Fe, wt.%	Al, wt.%	Gross Formula	XRD
DCS	650	I	41.0	34.4	33.2	-	CoFeC_3.7_N_0.04_	Co_0.5_Fe_0.5_
DCS + Al(OH)_3_	350	II	35.5	9.8	9.0	27.4	CoFeC_0.2_H_0.4_Al_7_O_x_(x > 1.2)	CoFe_2_O_4_, CoO, Fe_3_O_4_

**Table 2:** Particle size distribution.

	Sample I, μm	Sample II, μm
5% particle	1.37 μm	5.99 μm
25% particle	2.40 μm	14.69 μm
75% particle	3.89 μm	26.99 μm
100% particle	7.00 μm	109.88 μm

**Table 3:** Pore size distribution.

	V_des_ (d = 2–50 nm), sm^3^g^−1^	S (d = 2–50 nm), m^2^g^−1^	V_des_ (d > 50 nm), sm^3^g^−1^	S, (d > 50 nm), m^2^g^−1^	S_BET,_ m^2^g^−1^	V_des_, sm^3^g^−1^	Average Pore Size, nm
Sample **I**	0161	91.448	0.079	5.706	64.6	0.24	9.9
Sample **II**	0.200	230.854	0.014	1.145	218.5	0.21	3.7

**Table 4:** The main FTS parameters in presence of **I** and **II** catalysts.

Catalyst	Parameter	Temperature, °C						
		210	230	250	270	290	310	330
**I**	K_CO_,%	3	4	6	15	23	47	93
	S_C1–C4_	10	20	46	46	56	52	61
	S_C5+_	90	80	46	44	36	42	37
	S_Oxy_	0	0	8	10	8	6	2
	A, molCO·gMe^−1^ c^−1^	0.9	1.4	2.1	4.8	7.6	15.5	30.6
**II**	K_CO_,%	2	4	5	7	20	35	-
	S_C1–C4_	0	2	11	31	29	34	-
	S_C5+_	100	98	89	61	61	58	-
	S_Oxy_	0	0	0	8	10	8	-
	A, molCO·gMe^−1^ c^−1^	1.3	2.9	3.3	4.8	13.5	23.5	-

**Table 5:** Product composition of FTS at optimal synthesis temperature.

	*n*-Paraffins	Iso-Paraffins	Olefins	α_HC_	α_Oxy_
**I**	39.9	36.8	23.3	0.69	0.28
**II**	66.7	21.4	11.9	0.81	0.29

## Data Availability

Not applicable.

## Supplementary Materials

The following are available online. Figure S1: Powder X-ray diffraction patterns of [Co(NH_3_)_6_][Fe(CN)_6_] and catalytic compositions; Figure S2: IR spectra of [Co(NH_3_)_6_][Fe(CN)_6_] and catalytic compositions.

## Sample Availability

Samples of the compounds are not available from the authors.

## References

[1] Gholami Z., Tišler Z., Rubáš V. (2020). Recent advances in Fischer-Tropsch synthesis using cobalt-based catalysts: A review on supports, promoters, and reactors. Catal. Rev..

[2] De la Peña O’Shea V.A., Álvarez-Galván M.C., Campos-Martín J.M., Fierro J.L.G. (2007). Fischer–Tropsch synthesis on mono- and bimetallic Co and Fe catalysts in fixed-bed and slurry reactors. Appl. Catal. A Gen..

[3] Ma X., Sun Q., Cao F., Ying W., Fang D. (2006). Effects of the Different Supports on the Activity and Selectivity of Iron-Cobalt Bimetallic Catalyst for Fischer-Tropsch Synthesis. J. Nat. Gas Chem..

[4] Akbari M., Mirzaei A.A., Atashi H., Arsalanfar M. (2018). Effect of microemulsion parameters on product selectivity of MgO-supported iron–cobalt–manganese–potassium nanocatalyst for Fischer–Tropsch synthesis using response surface methodology. J. Taiwan Inst. Chem. Eng..

[5] Griboval-Constant A., Butel A., Ordomsky V.V., Chernavskii P.A., Khodakov A.Y. (2014). Cobalt and iron species in alumina supported bimetallic catalysts for Fischer–Tropsch reaction. Appl. Catal. A Gen..

[6] Zakharova E.Y., Makhaneva A.Y., Kulikova M.V., Chudakova M.V., Ivantsov M.I., Dementyeva O.D., Kuznetsov A.N. (2020). Metal-rich tellurides PdTe1−xBix as functional materials: Catalytic behavior in the Fischer–Tropsch synthesis and bonding analysis. Funct. Mater. Lett..

[7] Kulikova M.V., Ivantsov M.I., Zemtsov L.M., Chernavskii P.A., Karpacheva G.P., Bondarenko G.N., Khadzhiev S.N. (2015). Catalytic and magnetic properties of nanocomposites based on iron-containing polymer microspheres in Fischer-Tropsch synthesis. Pet. Chem..

[8] Kulikova M.V., Ivantsov M.I., Efimov M.N., Zemtsov L.M., Chernavskii P.A., Karpacheva G.P., Khadzhiev S.N. (2015). Formation features of composite materials containing cobalt nanoparticles active in Fischer-Tropsch synthesis. Eur. Chem. Bull..

[9] Potemkin D.I., Filatov E.Y., Zadesenets A.V., Gorlova A.M., Nikitina N.A., Pichugina D.A. (2020). A comparative study of CO preferential oxidation over Pt and Pt0.5Co0.5 nanoparticles: Kinetic study and quantum-chemical calculations. Mater. Lett..

[10] Potemkin D.I., Semitut E.Y., Shubin Y.V., Plyusnin P.E., Snytnikov P.V., Makotchenko E.V., Osadchii D.Y., Svintsitskiy D.A., Venyaminov S.A., Korenev S.V. (2014). Silica, alumina and ceria supported Au–Cu nanoparticles prepared via the decomposition of [Au(en)_2_]_2_[Cu(C_2_O_4_)_2_]_3_·8H_2_O single-source precursor: Synthesis, characterization and catalytic performance in CO PROX. Catal. Today.

[11] Potemkin D.I., Saparbaev E.S., Zadesenets A.V., Filatov E.Y., Snytnikov P.V., Sobyanin V.A. (2018). Preferential CO Oxidation on Bimetallic Pt_0.5_M_0.5_ Catalysts (M = Fe, Co, Ni) Prepared from Double Complex Salts. Catal. Ind..

[12] Khassin A., Pechenyuk S.I., Domonov D.P., Minyukova T.P., Chermashentseva G.K., Kustova G.N., Plyasova L.M. (2007). Catalytic properties OF bimetallic catalysts based on binary complexes of transition metals in fischer-tropsch synthesis. Chem. Sustain. Dev..

[13] Pechenyuk S.I., Domonov D.P., Gosteva A.N. (2018). Thermal decomposition of cationic, anionic and double complex compounds of 3d-metals. Russ. Chem. J..

[14] Pechenyuk S.I., Domonov D.P., Shimkin A.A., Semushina Y.P., Ivanov Y.V. (2017). Thermal behavior of binary complex compounds containing the hexacyanoferrate anion. Russ. J. Gen. Chem..

[15] Pechenyuk S.I., Domonov D.P., Gosteva A.N., Semushina Y.P., Shimkin A.A. (2018). Thermal behavior of double complexes [Co(NH_3_)_6_][Fe(CN)_6_] and [CO(en)_3_][Fe(CN)_6_]·2H_2_O. Izv. Vyss. Uchebnykh Zaved. Khimiya Khimicheskaya Tekhnologiya.

[16] Fu T., Jiang Y., Lv J., Li Z. (2013). Effect of carbon support on Fischer–Tropsch synthesis activity and product distribution over Co-based catalysts. Fuel Process. Technol..

[17] Lyu S., Peng B., Kuang T., Rappé K.G., Zhang Y., Li J., Wang L. (2019). Supported Cobalt Nanoparticles with a Single Active Phase for Fischer–Tropsch Synthesis. ACS Appl. Nano Mater..

[18] Vosoughi V., Badoga S., Dalai A.K., Abatzoglou N. (2016). Effect of Pretreatment on Physicochemical Properties and Performance of Multiwalled Carbon Nanotube Supported Cobalt Catalyst for Fischer–Tropsch Synthesis. Ind. Eng. Chem. Res..

[19] Kuang T., Lyu S., Liu S., Zhang Y., Li J., Wang G., Wang L. (2019). Controlled synthesis of cobalt nanocrystals on the carbon spheres for enhancing Fischer–Tropsch synthesis performance. J. Energy Chem..

[20] Lapidus A.L., Krylova A.Y. (2000). On mechanism of formation of liquid hydrocarbons from CO and H_2_ on cobalt catalysts. Ross. Khimicheskij Zhurnal (Zhurnal Ross. Khimicheskogo Obs. Im. D.I. Mendeleeva).

[21] Abbaslou R.M.M., Soltan J., Dalai A.K. (2010). Effects of nanotubes pore size on the catalytic performances of iron catalysts supported on carbon nanotubes for Fischer–Tropsch synthesis. Appl. Catal. A Gen..

[22] Maximov A.L., Kulikova M.V., Dementyeva O.S., Ponomareva A.K. (2020). Cobalt-Containing Dispersion Catalysts for Three-Phase Fischer–Tropsch Synthesis. Front. Chem..

[23] Cheng K., Virginie M., Ordomsky V.V., Cordier C., Chernavskii P.A., Ivantsov M.I., Paul S., Wang Y., Khodakov A.Y. (2015). Pore size effects in high-temperature Fischer–Tropsch synthesis over supported iron catalysts. J. Catal..

[24] Kulikova M., Chudakova M., Ivantsov M., Kuz’min A., Krylova A., Maksimov A. (2021). Properties of Cu-Co Composite Catalysts for Synthesis of Aliphatic Alcohols. J. Braz. Chem. Soc..

[25] Domonov D.P., Kuratieva N.V., Pechenyuk S.I. (2011). Structure and properties of double complex salts [Co(NH_3_)_6_][Fe(CN)_6_] and [Co(NH_3_)_6_] 2[Cu(C2O_4_)_2_]_3_. J. Struct. Chem..

[26] Pechenyuk S.I., Domonov D.P., Rogachev D.L., Belyavskii A.T. (2007). Anion effect on the thermolysis of double complexes [Co(NH_3_)_6_][Fe(CN)_6_] and [Co(NH_3_)_6_]4[Fe(CN)_6_]_3_. Russ. J. Inorg. Chem..

[27] Pechenyuk S.I., Semushina Y.P., Kadyrova G.I., Rogachev D.L., Kuz’mich L.F., Domonov D.P., Kalinnikov V.T. (2005). Synthesis and Properties of Double Complex Salts Containing the Cation [Co(NH_3_)_6_]^3+^. Russ. J. Coord. Chem..

