# Supplementary material for: Catalytic Activity of Thermolyzed [Co(NH3)6][Fe(CN)6] in CO Hydrogenation Reaction
Source: Molecules. 2021 Jun 22;26(13):3782. doi: 10.3390/molecules26133782 (PMC8270307; doi:10.3390/molecules26133782)
Supplement: Supplementary file 1 [file molecules-26-03782-s001.zip › molecules-1233299-supplementary.pdf]

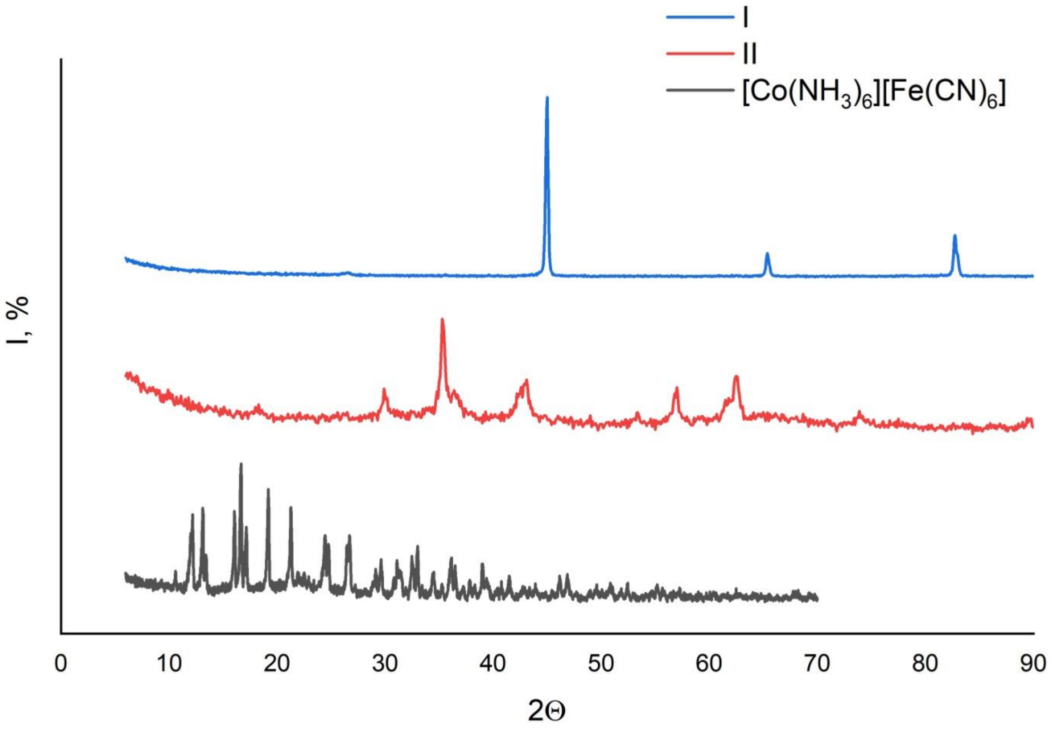

Figure S1. Powder X-ray diffraction patterns of $[Co(NH_3)_6][Fe(CN)_6]$ and catalytic compositions.

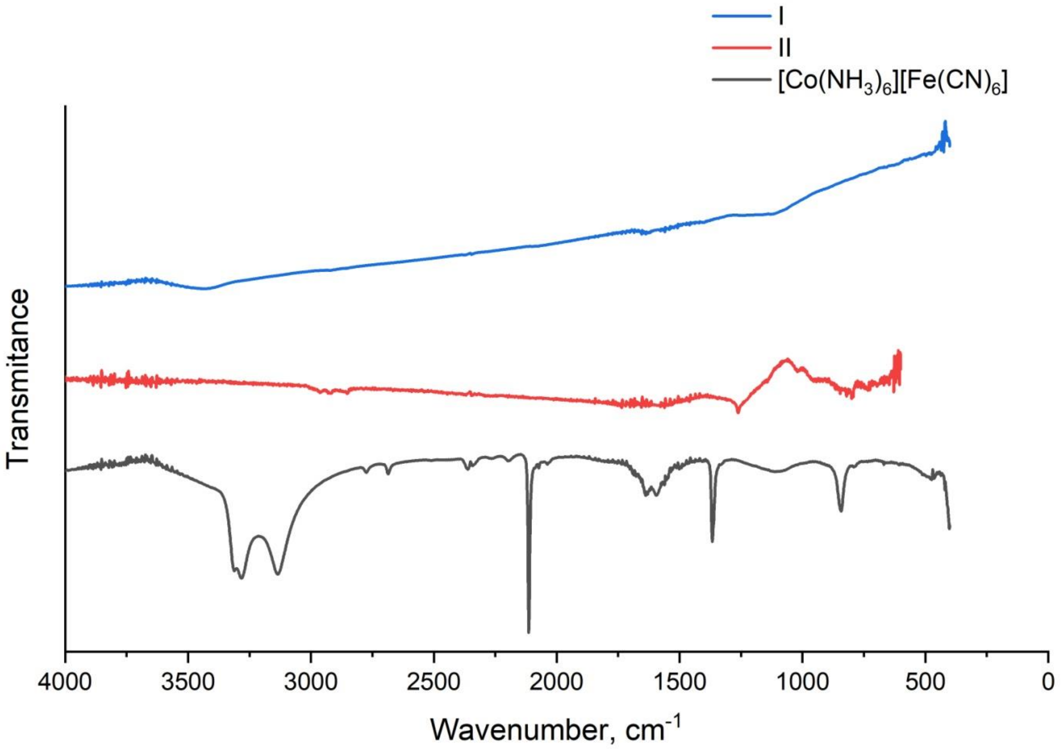

Figure S2. IR spectra of $[Co(NH_3)_6][Fe(CN)_6]$ and catalytic compositions.
